# Hybrid Majority Voting: Prediction and Classification Model for Obesity

**DOI:** 10.3390/diagnostics13152610

**Published:** 2023-08-07

**Authors:** Dahlak Daniel Solomon, Shakir Khan, Sonia Garg, Gaurav Gupta, Abrar Almjally, Bayan Ibrahimm Alabduallah, Hatoon S. Alsagri, Mandour Mohamed Ibrahim, Alsadig Mohammed Adam Abdallah

**Affiliations:** 1 Yogananda School of AI Computers and Data Sciences, Shoolini University, Solan 173229, India; 2 College of Computer and Information Sciences, Imam Mohammad Ibn Saud Islamic University (IMSIU), Riyadh 11432, Saudi Arabiasadigmo86@gmail.com (A.M.A.A.); 3 Department of Computer Science and Engineering, University Centre for Research and Development, Chandigarh University, Mohali 140413, India; 4 Department of Information System, College of Computer and Information Sciences, Princess Nourah Bint Abdulrahman University, Riyadh 11432, Saudi Arabia

**Keywords:** obesity, machine learning, majority voting, hybrid modeling, BMI

## Abstract

Because it is associated with most multifactorial inherited diseases like heart disease, hypertension, diabetes, and other serious medical conditions, obesity is a major global health concern. Obesity is caused by hereditary, physiological, and environmental factors, as well as poor nutrition and a lack of exercise. Weight loss can be difficult for various reasons, and it is diagnosed via BMI, which is used to estimate body fat for most people. Muscular athletes, for example, may have a BMI in the obesity range even when they are not obese. Researchers from a variety of backgrounds and institutions devised different hypotheses and models for the prediction and classification of obesity using different approaches and various machine learning techniques. In this study, a majority voting-based hybrid modeling approach using a gradient boosting classifier, extreme gradient boosting, and a multilayer perceptron was developed. Seven distinct machine learning algorithms were used on open datasets from the UCI machine learning repository, and their respective accuracy levels were compared before the combined approaches were chosen. The proposed majority voting-based hybrid model for prediction and classification of obesity that was achieved has an accuracy of 97.16%, which is greater than both the individual models and the other hybrid models that have been developed.

## 1. Introduction

**Figure 1:**
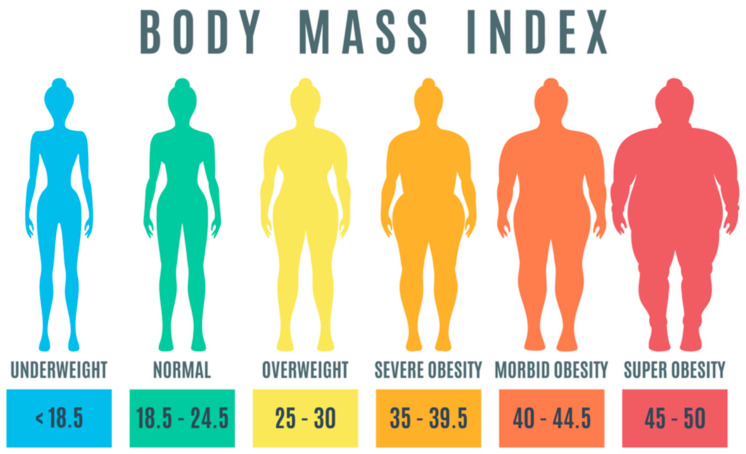
Obesity classification based on BMI [6].

Regardless of location, ethnicity, or socioeconomic class, obesity is a complicated, multifactorial disease that can affect both adults and children at any age. Additionally, obesity is one of the biggest risk factors for a variety of chronic diseases, including heart disease and cancer [1,2,3]. The World Health Organization (WHO) defines obesity as an abnormal or excessive deposition of fat that has the potential to severely impact health. Obesity can have a detrimental impact on health (BMI). The body mass index (BMI) of a person is calculated by taking their weight in kilograms and dividing it by the square of their height in meters. A BMI value of over 25 indicates that a person is obese, while a BMI value of over 30 indicates that they are overweight. Figure 1 provides a classification of obesity based on the ranges of BMI values. According to a report that was released by the WHO in 2017, it was shown that being overweight was directly responsible for the deaths of over 4 million people per year [4,5]. 

As the risk of obesity is associated with many other diseases, it is possible to address or alter some but not all of these risk factors. Finding modifiable risk factors for obesity at both the individual and population levels is essential for developing an effective strategy to reduce risk. Numerous studies have looked for better ways to predict obesity with the information already available. A variety of studies have attempted to analyze the data to form predictions by utilizing the benefits of data accessibility and availability. Because machine learning is one of the breakthrough innovations that deals with finding better and more detailed insights from data by implementing various mathematical and statistical approaches, several machine learning-based models have been proposed to predict and classify obesity at an early stage. A model of the predictability of the future can be constructed by using historical data in conjunction with a variety of machine learning methodologies. There models are used to forecast new data based on what they learned from studying historical data. 

The rest of this article is structured as follows: A thorough description of the literature review linked to this topic in Section 2 is provided. The procedures and resources employed are covered in Section 3. A description of the data source, data processing, machine learning techniques employed, and performance analysis matrix are all included. In Section 4, the experiments’ findings are presented, along with a comparison of the suggested method to those in Section 2 of the literature. The conclusion is presented in Section 5. 

## 2. Literature Review

Since health is the most important aspect of our day-to-day activities, there are various technological advancements and countless attempts to provide better health and safety. Machine learning is one of the most interesting research areas, and thus, it attracts many researchers to experiment with its methods on multi-criteria decision problems [7,8]. Machine learning helps to find better explanations from data and predict the future based on previously collected data. Many researchers are contributing towards using machine learning in critical diseases with high risk factors [9,10]. This section includes a brief review of the previous related papers on obesity prediction. 

Montanez et al. [11] provide a novel approach based on the analysis of genetic variants extracted from publicly available genetic profiles and the manually curated database. They identified 13 features for better prediction of obesity based on random forest dimension reduction. Then, using the selected features, they trained and tested different machine learning algorithms. By using quality measurement evaluation, the experimental study showed that the support vector machine (SVM) algorithm achieved a better accuracy. 

Singh et al. [12] presented an early-stage obesity prediction model for young people. The prediction model was prepared by using gender, weight, and BMI at the ages of 3, 5, 7, and 11 for training the model. Using the trained model, they forecasted the likelihood of obesity at age 14. During the training phase, they used many machine learning algorithms and conducted a comparative study to choose an algorithm with better performance. Based on the experimental findings, the multilayer perceptron neural network algorithm performed well, achieving 96% accuracy. 

Jindal et al. [13] presented a hybrid strategy for predicting obesity based on ensemble machine learning. The generalized linear model, random forest, and partial least squares were combined in the hybrid model. The model employs weight, height, age, gender, and BMI as its primary training and prediction characteristics. The hybrid model achieved an accuracy of 89.68%. As a further study, the researcher recommended including more than three algorithms for the ensemble-based hybrid approach.

Dugan et al. [14] presented a machine learning prediction model for early childhood. After the age of 2 years, the model is set up to use clinical data to make predictions for childhood obesity. The ID3 algorithm, which produces a tree based on the ID3 algorithm with no pruning, was used to analyze data from the Child Health Improvement through Computer Automation (CHICA) pediatric clinical decision support system. Predictions of childhood obesity using the proposed model were 85% accurate. 

Zheng et al. [15] presented an obesity prediction model for high school students. They used nine health-related behaviors from Tennessee’s 2015 Youth Risk Behavior Surveillance System (YRBSS) as input for the model. Binary logistic regression, an improved decision tree (IDT), a weighted k-nearest neighbor (KNN), and an artificial neural network (ANN) were the four improved machine learning models used in the study. According to the experiment, the accuracy of the modified decision tree and artificial neural network was 80.23% and 84.22%, respectively, while the accuracy of the k-nearest neighbors was 88.92%.

Taghiyev et al. [16] presented a hybrid approach using decision trees (DT) and logistic regression (LR) for identifying the cause and prediction of obesity. The hybrid model used two stages for better classifying the collected data: feature selection as the first stage and classification as the second stage. The study discovered that the obesity risk of women increases with increasing age, number of pregnancies, blood pressure, body weight, and blood glucose. The model presented in this paper performed with 91.4% accuracy.

Rodrigues et al. [17] provided a machine learning strategy for an obesity or overweight identification predictive model. The input data for the model had 16 features, which were based on the physical condition and eating habits of the person. By implementing and conducting an experiment on DT, SVM, k-NN, gaussian naive bayes, MLP, RF, gradient boosting, and extreme gradient boosting machine learning techniques, the study found that random forest showed better performance by achieving 78% accuracy.

Table 1 presents a comprehensive summary of the analyzed literature, providing a structured and systematic overview of the algorithmic methodologies employed, the feature extraction techniques utilized, the dataset selections made, and the corresponding model accuracy evaluations.

Studies that utilized methods of machine-learning-based prediction regarding the outcomes of an obesity prediction are included in this area. The fact that this topic has been discussed suggests that the outcomes of individual research are no longer reliable enough. There are several algorithms that perform better than others when compared to their overall performance. It is possible to acquire better results by analyzing and combining the results of several distinct machine learning approaches. According to the studies that were analyzed in this review of the relevant literature, when comparing strategies, it is necessary to consider a variety of methodologies. Additionally, the primary gap that needed to be filled, as well as the primary input for this study, was the suggestion of a hybrid model for improved prediction performance.

## 3. Materials and Methods

This section discusses the materials and methods that were utilized for this research. The first subsection contains specific information regarding the data sources; the second subsection discusses the data processing procedures that were carried out to prepare the dataset; and the final subsection explains the machine learning algorithms that were implemented.

### 3.1. Data Source

**Figure 2:**
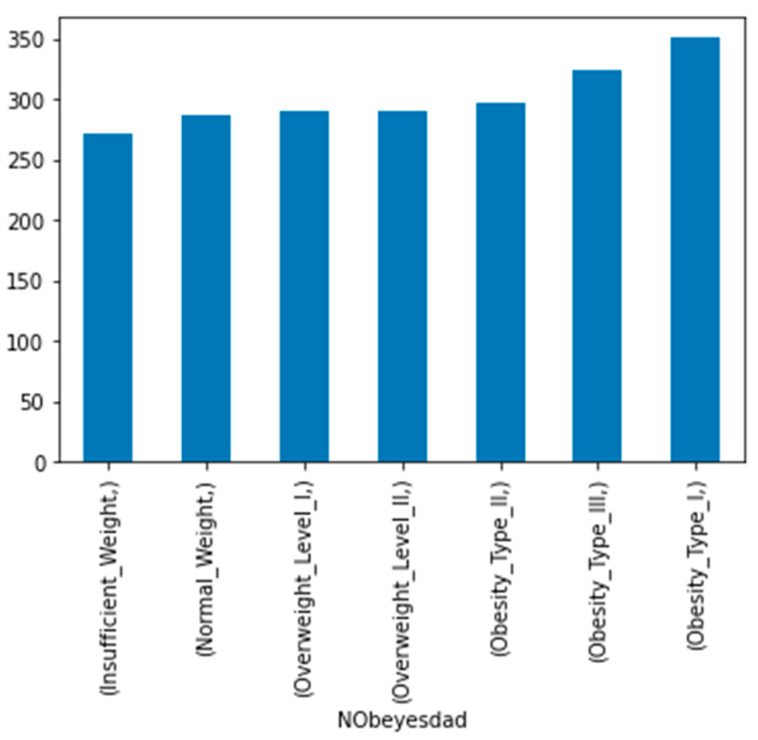
Classification of independent variables.

The dataset that was utilized in this study was acquired from the UCI repository, which is one of the most popular public machine learning data repositories [18], and the public dataset that was utilized for this research has 2111 records under 17 features that were collected from Mexico, Peru, and Colombia. The dataset consisted of data based on eating habits and physical conditions [19]. The records were labeled with the class variable NObesitydad, which indicates the level of obesity using seven classifications. Table 2 shows detailed information about the attributes with their descriptions and variable type, and Figure 2 shows the seven different classifications based on the dependent variable. 

### 3.2. Data Processing

**Figure 3:**
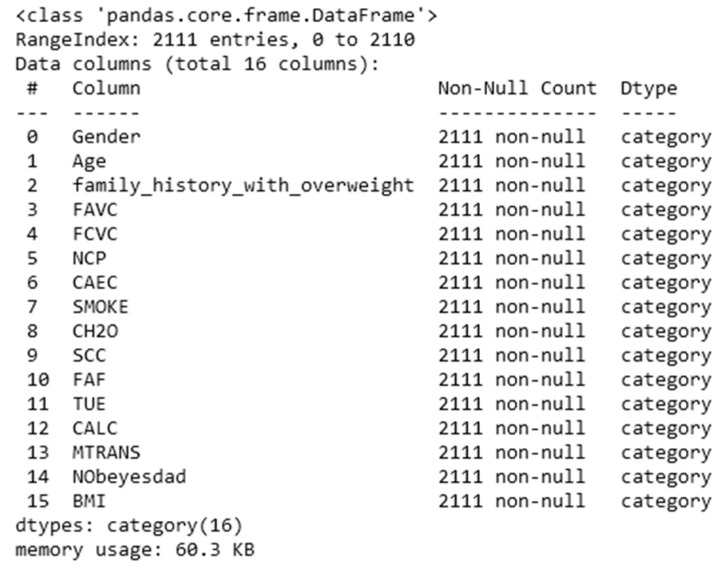
Results of missing value check.

The term “data processing” refers to the process of gathering data for analyzing digital data to draw conclusions. Information processing, of which data processing is a subset, refers to any action taken on data that results in observable changes to the data [20]. The first stage of the data processing procedure, known as data extraction, consisted of the retrieval of data from a comma-separated values file (CSV) and its subsequent placement in a data frame. The data extraction was followed by data cleaning, which deals with the data being reviewed for null values and outliers, and then steps were taken to rectify the same. The results of the missing value check are shown in Figure 3. 

**Figure 4:**
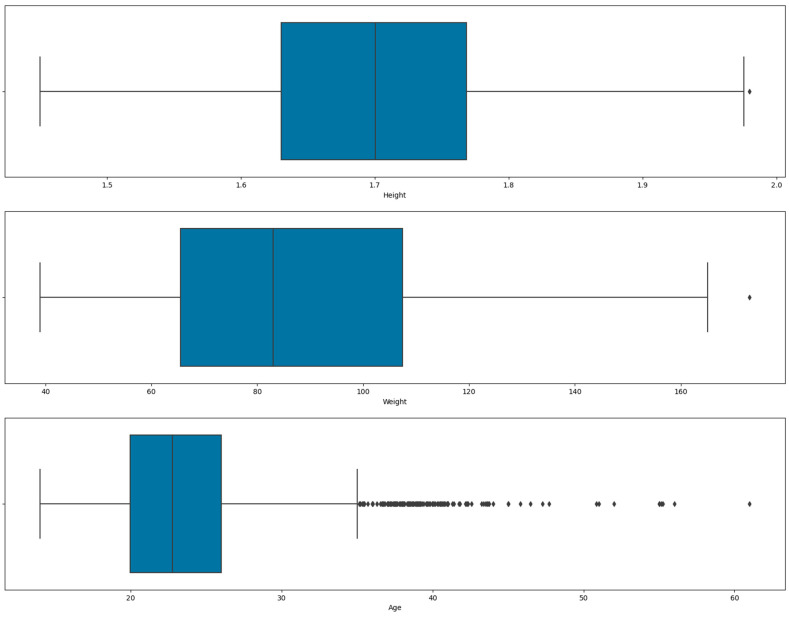
Box plot representation of height, weight, and age outliers.

Figure 4 shows the outliers representation for height, weight, and age using a box plot. 

**Figure 5:**
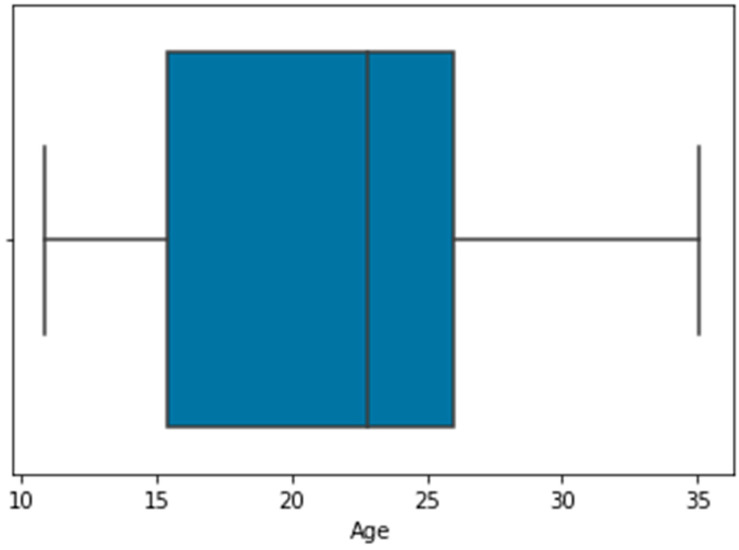
After removal of outliers.

As shown in Figure 3, the age attribute had outliers. The total number of outliers detected for age was 168. Figure 5 shows the results after the removal of outliers for age.

**Figure 6:**
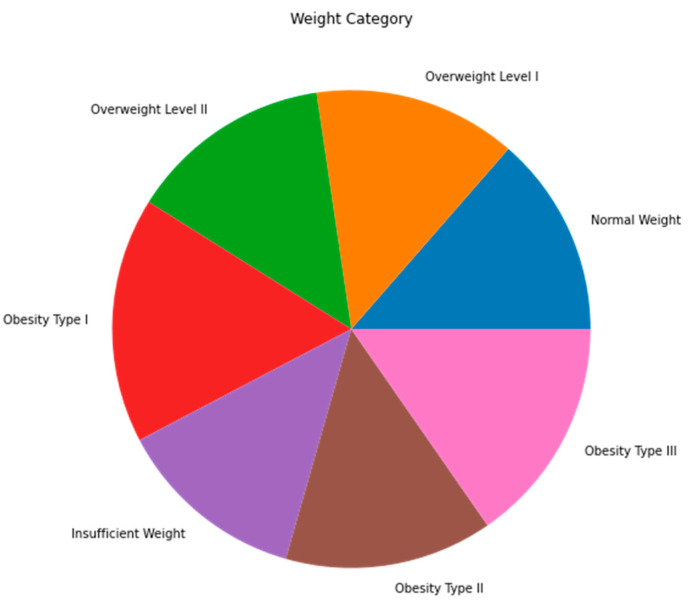
Target class data balance.

Figure 6 shows that the target data in the dataset was balanced.

**Figure 7:**
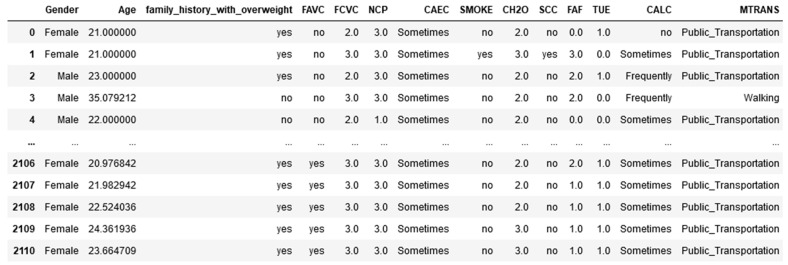
Before data transformation.

**Figure 8:**
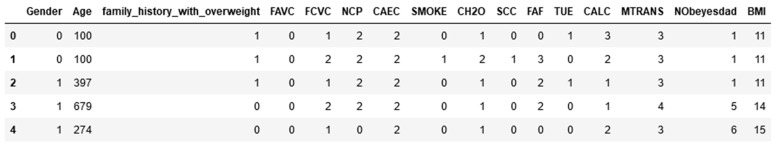
After data transformation.

Following data cleaning, data transformation was carried out. A whole set of values for a given attribute was mapped to a new set of replacement values in a process known as data transformation so that the old values could be associated with one of the new values. Figure 7 and Figure 8 show before and after data transformations were performing on the dataset, respectively. 

### 3.3. Machine Learning Techniques

The practice of teaching computer systems to learn and make predictions or judgments based on data is known as machine learning or deep learning. Because sensitive data must be accessed during the training phase for machine learning and deep learning, privacy issues arise. Machine learning and deep learning have become more common in a variety of industries and applications by ensuring privacy and security. This is because of their capacity to analyze massive amounts of data and extract insightful information [21,22,23,24,25,26].

An ensemble is a group of districts and numerous models in which each model offers a forecast, and the final prediction is established by a majority vote among the models. An ensemble is also sometimes referred to as a supermodel. The integration of multiple classifiers is accomplished with the objective of producing results that are superior to those produced by a single classifier. Ensemble methods that utilize voting-based aggregation, like voting classifiers, can enhance the overall performance of the ensemble by reducing individual model biases and errors. They are particularly useful when the individual models excel at different aspects or capture different patterns in the data. However, it is important to note that voting-based ensembles are most effective when the individual models are diverse and exhibit complementary strengths, rather than being highly correlated or similar.

It is a strategy that can be used to increase the accuracy of the classifier, and it is an efficient strategy for meta categorization that combines ineffective learners with effective learners to improve the effectiveness of the ineffective learners [23]. After building and comparing seven alternative algorithms, this study employed ensemble learning to combine three machine learning models. The seven machine learning models that are compared in this research are

⮚ Gradient boosting classifier;
⮚ XGB classifier;
⮚ Multilayer perceptron;
⮚ K-nearest-neighbor classifier;
⮚ Logistic regression;
⮚ Naïve Bayes classifier;
⮚ Random forest classifier;
⮚ Decision tree.

## 4. Results and Discussion

This section aims to present and discuss the findings of the study in three distinct sections. Each section focuses on a specific aspect of the research and provides a comprehensive analysis of the results.

The first section is dedicated to describing the results of the exploratory investigation conducted on the dataset used in the study. This exploratory analysis involves a detailed examination of the dataset’s characteristics, distribution, and patterns. It aims to provide insights into the data’s composition and identify any notable trends or relationships. By presenting the findings of this exploratory investigation, readers gain a deeper understanding of the dataset and the factors influencing obesity prediction.

The second section focuses on presenting the numerical performance indicators of the approaches and methodologies incorporated in the study, including the proposed hybrid model. These performance indicators, such as accuracy rates, precision, recall, or F1 scores, provide quantitative measures of the models’ predictive capabilities. By presenting these numerical results, the section offers a comprehensive evaluation of the individual approaches and the proposed hybrid model, allowing readers to assess their effectiveness in accurately predicting obesity.

The last section of the study presents a comparative study of the proposed model. This section aims to compare the performance of the proposed hybrid model with other existing models or approaches in the field. By conducting this comparative analysis, the study highlights the strengths and advantages of the proposed model over other approaches, demonstrating its potential as an innovative and effective solution for obesity prediction. This comparative study provides valuable insights into the unique contributions and advancements made by the proposed model, setting it apart from existing approaches.

By presenting and discussing the findings across these three sections, the study provides a comprehensive overview of the research results. It allows readers to gain a holistic understanding of the exploratory investigation, the numerical performance of the approaches, methodologies, and proposed hybrid model, as well as the comparative study. This comprehensive analysis contributes to the overall significance and impact of the research, providing valuable insights for researchers, practitioners, and stakeholders interested in obesity prediction and machine learning applications in healthcare.

### 4.1. Result of Exploratory Analysis

To gain a deeper understanding of the dataset, exploratory data analysis (EDA) was conducted on the dataset sourced from the UCI machine learning repository. This process involved examining and analyzing various characteristics and features present in the dataset. The findings from these analyses are presented in detail in the following sections.

**Figure 9:**
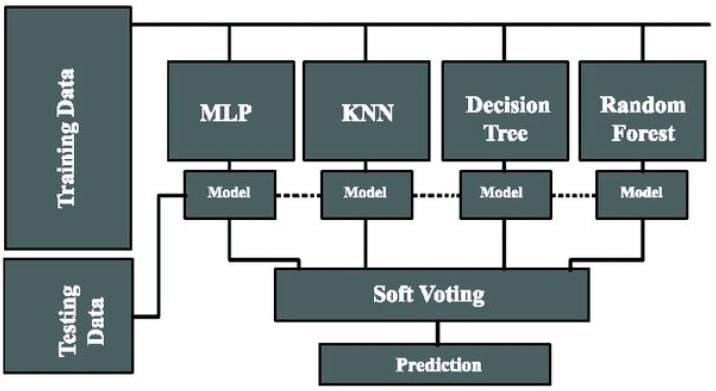
Voting classifier architecture [27].

One crucial aspect of the EDA was to explore the distribution and patterns of specific variables within the dataset. This allowed us to uncover insights regarding the prevalence and relationships of certain attributes. Among these variables, Figure 9 provides a visual representation of the data distribution outcomes for several key factors, including smoking, gender, family history, alcohol usage, mode of transportation, and frequency of consumption of high-calorie foods.

**Figure 10:**
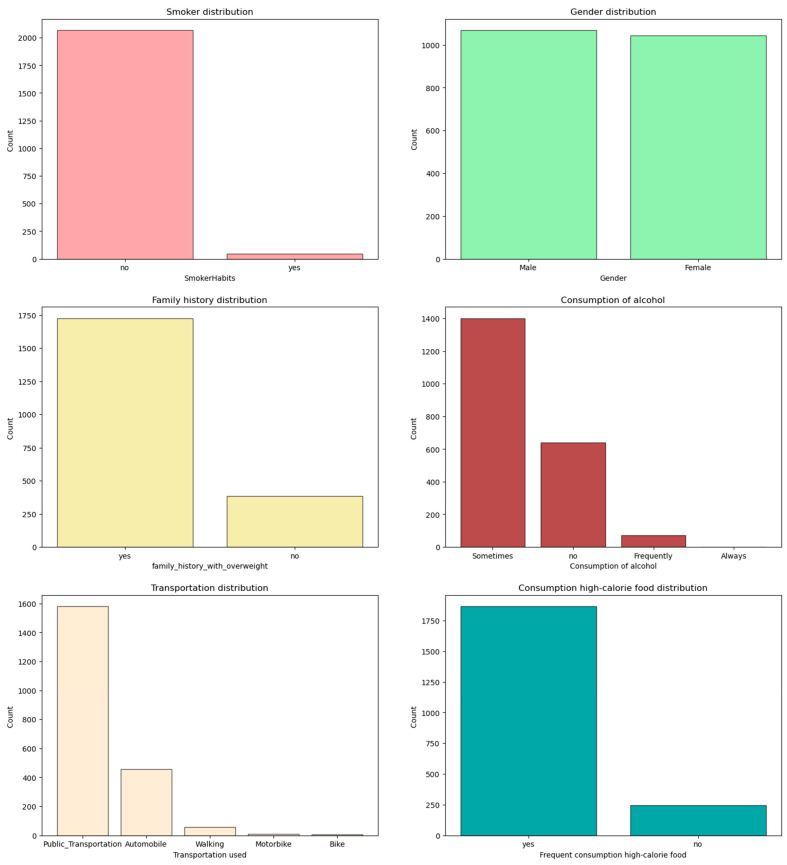
Data distribution outcomes.

By examining the distribution outcomes in Figure 10, we can observe the relative frequencies or proportions of each category within the respective variables. This graphical representation helps us identify any imbalances or biases present in the dataset. For instance, we can ascertain whether the dataset contains a significant proportion of smokers or if there is an equal distribution of gender representation.

Furthermore, Figure 10 sheds light on the prevalence of specific characteristics related to obesity. It allows us to assess the significance of factors such as family history, alcohol usage, mode of transportation, and the frequency of high-calorie food consumption in the dataset. This information is crucial for understanding the potential influence of these variables on the prediction and classification of obesity.

**Figure 11:**
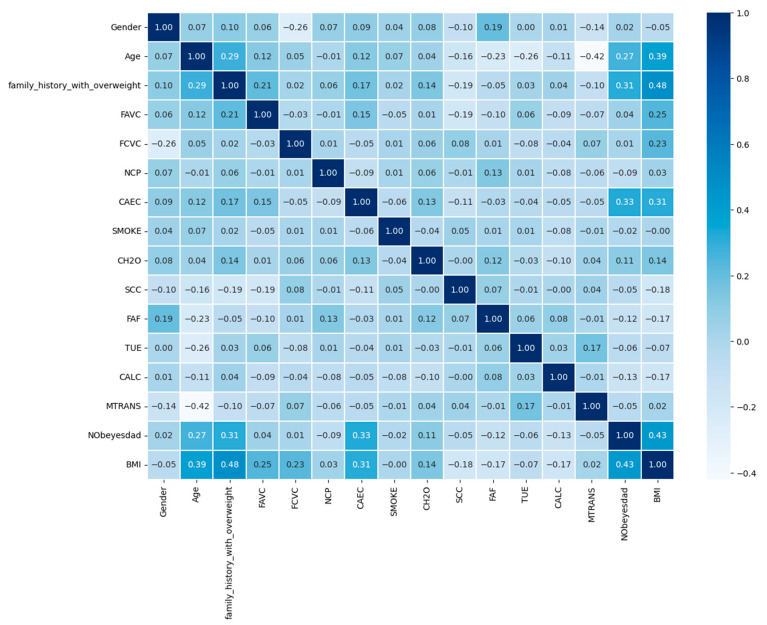
Heatmap of correlation among the features.

A heatmap of values and associations is shown in Figure 11. Each colored cell shows a relationship between two qualities and their linked values, with the relationship indicated by the color of the cell. A correlation value less than zero suggests a negative link, whereas a correlation value of zero shows no association.

### 4.2. Result of Machine Learning Analysis

This section aims to provide a detailed description of the performance of the suggested hybrid model for predicting the outcome of majority voting. It will also include the results obtained from the individual machine learning approaches that were applied. To facilitate comparison, Table 3 presents the levels of accuracy achieved by each machine learning model as well as the accuracy of the suggested hybrid model. To evaluate the performance of the hybrid model, we conducted experiments using the selected machine learning techniques, namely gradient boosting classifier, XGB classifier, and multilayer perceptron classifiers. Each of these models was trained and tested on the designated dataset, and their individual accuracy rates were recorded.

Table 3 provides a clear overview of the accuracy levels achieved by the individual machine learning models, as well as the accuracy of the hybrid model. By comparing the results, we can determine the effectiveness of the hybrid model in improving the accuracy of obesity prediction compared to the individual models. As indicated in Table 3, the suggested hybrid model outperforms the findings obtained from the individual machine learning approaches. It demonstrates a higher level of accuracy, showcasing the added value of combining multiple models through majority voting to enhance prediction performance. The comparative analysis presented in Table 3 allows for a quantitative assessment of the proposed hybrid model’s superiority over the individual machine learning models. It highlights the advantages of leveraging ensemble learning techniques to consolidate predictions from multiple models and achieve improved accuracy. Additionally, this information provides valuable insights into the potential of the hybrid model in real-world applications. The higher accuracy rate obtained by the hybrid model suggests its potential to generate more accurate predictions and classifications related to obesity, which can be beneficial for medical professionals, researchers, and policymakers working in the field. In this study, a one-stage aggregation method was utilized, but, on the other hand, in a two-stage aggregation approach, multiple machine learning methods are combined to improve predictive performance. In the first stage, diverse base models are selected and trained independently. This allows each model to capture different aspects of the data and contribute unique insights. Hyperparameter tuning and feature selection can be applied to optimize the performance of these models. In the second stage, the predictions of the base models are aggregated using techniques such as voting, weighted averaging, stacking, or an ensemble of ensembles. This aggregation process combines the strengths of individual models and produces a final prediction that benefits from their collective knowledge. The aggregated model is then evaluated to assess its performance, and further fine-tuning of the aggregation technique or base model weights can be conducted if necessary. The two-stage approach leverages model diversity and aims to achieve superior predictive accuracy compared to using a single model [28,29].

### 4.3. Comparative Analysis of Results

Performing a comparative analysis is crucial to showcase the potential of the proposed hybrid model. Given that obesity prediction is a popular and extensively studied topic among machine learning researchers, there are numerous models that have been proposed for this purpose. To provide a comprehensive review of the existing literature, Table 4 will be utilized to compare the proposed hybrid model with other relevant studies.

By conducting a thorough literature review, we have identified several existing models for the prediction of obesity. These models serve as benchmarks against which the performance and effectiveness of our proposed hybrid model can be assessed. Table 4 presents a summary of these studies, highlighting key aspects such as the employed methodologies, datasets used, and achieved accuracy rates.

The purpose of this comparative analysis is to provide insights into the strengths and weaknesses of the existing models and to position our proposed hybrid model in relation to them. By evaluating the performance metrics and accuracy rates of each model, we can gain a better understanding of the advancements and limitations in the field of obesity prediction using machine learning techniques. Moreover, the proposed model can be efficiently used for other applications of deep learning and machine learning other than obesity [30,31,32,33,34,35,36,37,38].

## 5. Conclusions

In conclusion, this study has demonstrated the effectiveness of a hybrid model for improving the accuracy and classification capabilities of machine learning in the context of obesity. By combining multiple machine learning approaches using voting-based ensemble learning, we were able to enhance the performance of the models by incorporating the outputs from various models as inputs for the hybrid machine learning model. Initially, we conducted an evaluation of seven distinct machine learning techniques individually to assess their predictive capabilities. Following this analysis, we identified the gradient-boosting classifier, XGB classifier, and multilayer perceptron classifiers as the most promising techniques for our hybrid prediction and classification model.

The hybrid model was created by integrating three selected machine learning techniques, utilizing the concept of ensemble learning. Ensemble learning combines predictions from multiple models to make a final prediction, often through a majority vote. In this case, a majority voting-based approach was employed to combine the outputs of the three classifiers. The resulting hybrid model, based on majority voting, achieved an impressive accuracy rate of 97.16%. This demonstrates the model’s ability to generate accurate predictions and classifications regarding obesity by leveraging the combined insights from the integrated machine learning techniques. The research highlights the potential of hybrid models for improving the performance of machine learning algorithms, particularly in complex tasks like obesity prediction and classification. The successful implementation and evaluation of the hybrid model offer valuable insights into the development of more robust and accurate machine learning systems for addressing obesity-related challenges.

In this research endeavor, an expansion of machine learning methodologies is planned to be incorporated into the hybrid model. Furthermore, diverse data partitioning and individual model training techniques will be explored and implemented prior to the construction of the hybrid model. The ensuing comparison will encompass a range of metrics, including time efficiency assessments, aimed at providing a comprehensive and insightful evaluation of the model’s performance.

## Figures and Tables

**Table 1:** Summary of literature review.

Article	Title	Algorithm	Feature Used	Dataset	Accuracy
Montanez et al. [11]	“Machine Learning Approaches for the Prediction of Obesity using Publicly Available Genetic Profiles”	SVM	13	6622	90.5%
Singh et al. [12]	“Machine Learning Approach for the Early Prediction of the Risk of Overweight and Obesity in Young People”	Multilayer perceptron neural network	4	11,110	96%
Jindal et al. [13]	“Obesity Prediction Using Ensemble Machine Learning Approaches”	Ensemble machine learning	5	600	89.68%
Dugan et al. [14]	“Machine Learning Techniques for Prediction of Early Childhood Obesity”	Decision tree (ID3)	167	7519	85%
Zheng et al. [15]	“Using Machine Learning to Predict Obesity in High School Students”	k-NN	9	5227	88.82%
Taghiyev et al. [16]	“A Hybrid Approach Based on Machine Learning to Identify the Causes of Obesity”	Decision trees (DT) and Logistic regression (LR)	26	500	91.4%
Rodriguez et al. [17]	“Machine learning techniques to predict overweight or obesity”	Random forest	16	2111	78%

**Table 2:** Detail of features on the dataset.

S. No	Feature Name	Description	Variable Type	Category	Range
1	Gender	Gender of the person	Categorical	Respondent characteristics	Male or Female
2	Age	Age in years	Integer	Respondent characteristics	14 to 61
3	Hight	Hight in meters	Float	Respondent characteristics	1.45 to 1.98
4	Weight	Weight in kilograms	Float	Respondent characteristics	39 to 173
5	Family history with overweight	Family history of obesity	Categorical	Respondent characteristics	Yes or no
6	FAVC	High caloric food consumption	Categorical	Eating habit	Yes or no
7	FCVC	Frequency of vegetable intake	Ordinal	Eating habit	1 to 3
8	NCP	Number of primary meals	Ordinal	Eating habit	1 to 4
9	CAEC	Consumption of food	Ordinal	Eating habit	No, Sometimes, Frequently, Always
10	SMOKE	Smoking habit	Categorical	Physical condition	Yes or No
11	CH2O	Water consumption per day	Ordinal	Eating habit	1 to 3
12	SCC	Tracking calorie consumption	Categorical	Physical condition	Yes or no
13	FAF	Frequency of physical activity	Ordinal	Physical condition	0 to 3
14	TUE	Time spent on electronic gadgets	Ordinal	Physical condition	0 to 2
15	CALC	Alcohol consumption	Categorical	Eating habit	No, Sometimes, Frequently, Always
16	MTRANS	Type of transportation used	Categorical	Physical condition	Public, Walking, Automobile, Motorbike,
17	NObeyesdad	BMI	Categorical	Target Variable	Insufficient, Normal, Overweight Level I, Overweight Level II, Obesity Level I, Obesity Level II, Obesity Level III

**Table 3:** Model accuracy comparison.

Model	Accuracy
SVM	86.75%
GaussianNB	88.17%
kNN	78.23%
LR	86.91%
DT	94.95%
RF	91.95%
eXtreme Gradient Boosting	96.37%
Gradient Boost	96.06%
XGBoost	96.06%
MLP	93.38%
Proposed hybrid model	97.16%

**Table 4:** Comparative analysis of comparative studies.

Article	Algorithm	Feature Used	Dataset	Accuracy
Montanez et al. [11]	SVM	13	6622	90.5%
Singh et al. [12]	Multilayer perceptron neural network	4	11,110	96%
Jindal et al. [13]	Ensemble machine learning	5	600	89.68%
Dugan et al. [14]	Decision tree (ID3)	167	7519	85%
Zheng et al. [16]	k-NN	9	5227	88.82%
Taghiyev et al. [16]	Decision trees (DT) and logistic Regression (LR)	26	500	91.4%
Rodriguez et al. [17]	Random forest	16	2111	78%
This work	Majority voting-based hybrid modeling	16	2111	97.16%

## Data Availability

The data used in this article can be obtained from the corresponding author on request.
